# Chronic Kidney Disease-Associated Pruritus in Hemodialysis: Unraveling Mechanisms and Emerging Therapeutic Targets—A Systematic Review

**DOI:** 10.3390/ijms27020851

**Published:** 2026-01-15

**Authors:** Fasie Dragos, Suliman Ioana Livia, Panculescu Florin Gabriel, Cimpineanu Bogdan, Alexandru Andreea, Alexandrescu Luana, Alexandrescu Maria Daria, Popescu Stere, Enache Florin-Daniel, Manac Iulian, Mihai Lavinia Mihaela, Popa Marius Florentin, Tudor Iuliana-Cezara, Nitu Radu Adrian, Chisnoiu Tatiana, Cozaru Georgeta Camelia, Hangan Tony, Tuta Liliana-Ana

**Affiliations:** 1 “Sfantul Apostol Andrei” Emergency County Clinical Hospital, 900591 Constanta, Romania; dragosfasie@yahoo.com (F.D.); gabriel.panculescu@yahoo.ro (P.F.G.); alexandra_med16@yahoo.com (A.A.); luana.alexandrescu@365.univ-ovidius.ro (A.L.); stelu_popescu@yahoo.com (P.S.); florin.enache@365.univ-ovidius.ro (E.F.-D.); iulian.manac@gmail.com (M.I.); m.mihaelalavinia@yahoo.ro (M.L.M.); marius_popa2005@yahoo.com (P.M.F.); radu_nitzu@yahoo.com (N.R.A.); tatiana_ceafcu@yahoo.com (C.T.); tony@medcon.ro (H.T.); tuta.liliana@univ-ovidius.ro (T.L.-A.); 2 Faculty of Medicine, “Ovidius” University of Constanta, 900470 Constanta, Romania; dr.tudor.cezara@gmail.com; 3 Faculty of Medicine, Titu Maiorescu University, 040051 Bucharest, Romania; alexandrescu_daria@yahoo.com; 4 Center for Research and Development of the Morphological and Genetic Studies of Malignant Pathology (CEDMOG), “Ovidius” University of Constanta, 900591 Constanta, Romania; drcozaru@yahoo.com; 5 Academy of Romanian Scientist, 3 Ilfov Street, 050044 Bucharest, Romania

**Keywords:** chronic kidney disease, uremic pruritus, hemodialysis, κ-opioid receptor, uremic toxins, neurocutaneous pathways

## Abstract

This systematic review examines chronic kidney disease-associated pruritus (CKD-aP) as a complex clinical manifestation in patients undergoing hemodialysis. Traditionally considered a secondary symptom of end-stage renal disease, emerging evidence now positions CKD-aP as a multidimensional disorder with substantial pathogenic influence on patient outcomes. Using the PRISMA 2020 methodology, we critically evaluated 54 peer-reviewed studies published between 2020 and 2025. Our synthesis highlights a convergence of five mechanistic frameworks underpinning CKD-aP: elevated levels of uremic toxins originating from gut microbial dysbiosis, immune activation driven by IL-31 and other pro-inflammatory cytokines, heightened peripheral and central neural sensitization, dysregulation of endogenous opioid receptor pathways favoring μ-receptor activation, and xerosis-related epidermal barrier dysfunction. These mechanisms contribute to a systemic cycle of microinflammation, pruritogenic signaling, and neural hyperexcitability. We also identified and compared validated assessment tools—including the NRS, VAS, Skindex-10, and the UP-Dial scale—that facilitate standardized quantification of disease burden. While available treatments such as gabapentinoids and phototherapy offer partial relief, targeted therapies—including κ-opioid receptor agonists—represent a major advancement, although long-term effectiveness and accessibility remain under investigation. Growing scientific consensus establishes CKD-aP as a priority therapeutic target in hemodialysis care, underscoring the need for integrated, mechanism-based management strategies to improve quality of life and clinical outcomes. This work represents a narrative systematic review, integrating evidence from mechanistic, translational, and clinical studies to critically examine the biological pathways underlying CKD-associated pruritus.

## 1. Introduction

Chronic kidney disease-associated pruritus (CKD-aP), also referred to as uremic pruritus, is a frequent and debilitating complication in patients with advanced chronic kidney disease undergoing maintenance hemodialysis. Despite improvements in renal replacement therapies, a substantial proportion of patients continue to experience persistent itching, often with moderate-to-severe intensity, leading to sleep disturbance, psychological distress, impaired social functioning, and reduced health-related quality of life. Importantly, epidemiological studies have demonstrated associations between severe pruritus and increased morbidity and mortality, underscoring its clinical relevance beyond symptomatic discomfort [1,2].

Clinically, CKD-aP typically presents as chronic, generalized pruritus in the absence of primary dermatologic disease. Although xerosis is commonly observed in hemodialysis populations, skin dryness alone does not adequately explain the occurrence or severity of itch, suggesting a more complex systemic pathophysiology. Conventional antipruritic therapies, including antihistamines, are frequently ineffective, further supporting the notion that CKD-aP is predominantly non-histaminergic in nature.

Accumulating evidence indicates that CKD-aP arises from the interaction of multiple biological pathways involving immune dysregulation, neural sensitization, endogenous opioid receptor imbalance, and cutaneous barrier dysfunction. Systemic microinflammation and the accumulation of uremic metabolites are thought to act as upstream drivers, promoting pruritogenic cytokine release and altered neural signaling. Among these pathways, opioid receptor dysregulation has emerged as a particularly compelling mechanism, supported by recent interventional data demonstrating the efficacy of κ-opioid receptor agonists. Nevertheless, mechanistic certainty varies across proposed pathways, and several remain supported primarily by associative or preclinical evidence [3,4].

Given this mechanistic complexity, CKD-aP is increasingly recognized as a neuro–immune–cutaneous disorder rather than a purely dermatologic symptom. A clearer understanding of the hierarchical relationships and interactions among these pathways is essential for advancing targeted, mechanism-based therapies and improving patient outcomes.

Accordingly, this narrative systematic review aims to critically synthesize recent evidence on the molecular and pathophysiological mechanisms underlying CKD-aP in hemodialysis patients. In addition, it highlights validated assessment tools and emerging therapeutic strategies through a translational lens, emphasizing areas of robust evidence, unresolved mechanistic gaps, and future directions for research and clinical management [5,6].

## 2. Methods

### 2.1. Search Strategy

A structured literature search was conducted from January 2020 to June 2025 in PubMed/MEDLINE, Scopus, Web of Science Core Collection, and Cochrane Library databases. Search terms included combinations of:

“uremic pruritus,” “chronic kidney disease,” “hemodialysis,” “itch,” “pathophysiology,” “assessment,” and “quality of life.” Boolean operators (“AND,” “OR”) were used to refine results. This review was prospectively registered on the Open Science Framework (OSF) under the registration number 10.17605/OSF.IO/YE5P2. No major deviations from the registered protocol occurred [7].

Database-specific strategies combined controlled vocabulary (where applicable) and free-text keywords. In PubMed/MEDLINE, both MeSH terms and Title/Abstract fields were used; in Scopus and Web of Science, searches were performed in Title/Abstract/Keywords fields; in the Cochrane Library, searches were performed in Title/Abstract/Keywords. Filters were restricted to English language and publication years 2020–2025. No geographic restrictions were applied [8,9].

### 2.2. Inclusion and Exclusion Criteria

Included studies were:

- peer-reviewed English-language articles,
- conducted in adult CKD or hemodialysis populations,
- focused on pathophysiology, epidemiology, assessment, or management of CKD-aP.

Exclusion criteria:

- pediatric studies,
- case reports without clinical or mechanistic relevance,
- non-peer-reviewed materials (conference abstracts, letters without data).

The review primarily focused on adult patients undergoing hemodialysis with CKD-associated pruritus. Outcomes of interest included mechanistic insights, pruritus severity, quality-of-life impact, and therapeutic targets. Studies involving non-dialysis CKD populations were considered only when providing relevant mechanistic information.

### 2.3. Data Extraction and Synthesis

Titles and abstracts were screened independently by two reviewers to identify potentially eligible studies. Full-text articles were subsequently assessed independently by the same reviewers against the predefined inclusion/exclusion criteria. Discrepancies at any stage were resolved by consensus; when needed, a third reviewer adjudicated. Formal inter-rater agreement statistics were not calculated due to the narrative mechanistic scope and heterogeneity of study types; however, eligibility criteria were applied consistently using a standardized screening approach.

Results were synthesized narratively due to heterogeneity of study designs.

Mechanistic pathways were categorized according to the level of available evidence (human molecular data, clinical correlations, or preclinical models) to facilitate critical interpretation [5,6].

### 2.4. Risk of Bias and Data Management

Two reviewers independently screened and extracted data using a standardized Excel form capturing study design, population, interventions, and outcomes. Disagreements were resolved by discussion. As this was a narrative systematic review, no meta-analysis or quantitative pooling was performed. Risk of bias was assessed narratively based on study design and reporting clarity rather than formal tools. Publication bias was not formally evaluated [10].

Given the mechanistic and translational focus of this review and the heterogeneity of included study designs, a formal quantitative risk-of-bias assessment was not performed. Instead, studies were appraised narratively based on design (human vs. animal studies, interventional vs. observational), methodological transparency, and relevance to molecular or pathophysiological mechanisms. Mechanistic hypotheses were interpreted with caution when supported primarily by associative or preclinical data.

Formal risk-of-bias assessment using standardized tools (e.g., RoB 2, ROBINS-I, COSMIN) was not performed, as the included literature encompassed highly heterogeneous study designs, including mechanistic studies, observational cohorts, randomized trials, and validation studies of assessment instruments. Instead, a narrative critical appraisal was applied, with attention to study design, consistency of findings, and relevance to human mechanistic evidence. This approach aligns with the exploratory and integrative aims of the review.

### 2.5. Flow of Studies

**Figure 1:**
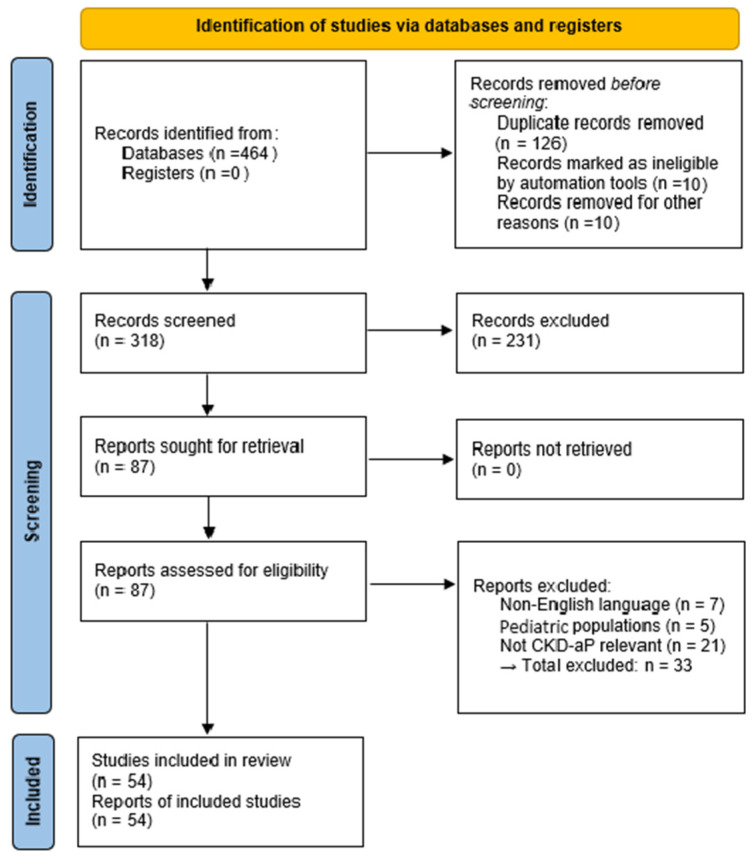
PRISMA 2020 Flow Diagram.

The search retrieved 452 records (PubMed n = 210, Scopus n = 120, Web of Science n = 102, Cochrane n = 20). After removing duplicates, 318 abstracts were screened; 87 full-texts were reviewed; and 54 studies met the inclusion criteria (Figure 1, PRISMA flow diagram) and PRISMA statement (see Supplementary Materials). Full-text articles were excluded primarily due to irrelevance to CKD-associated pruritus mechanisms, non-adult populations, or lack of mechanistic or clinical relevance. Given the narrative synthesis approach, exclusions were not tabulated individually but are reflected in the PRISMA flow diagram.

## 3. Results and Discussion

CKD-associated pruritus (CKD-aP) continues to represent a major clinical challenge with significant health-related and prognostic implications. Across the studies included in this review, prevalence ranges from 31% to 64% among patients receiving maintenance hemodialysis [11]. Severe pruritus is strongly associated with sleep disturbance, fatigue, decreased social functioning, mood impairment, and reduced quality of life [12]. In addition, CKD-aP has been linked to increased hospitalization events and all-cause mortality, demonstrating its systemic impact beyond cutaneous discomfort [13]. These findings support the conceptualization of CKD-aP as a complex disorder with multifaceted pathophysiology and substantial clinical burden.

### 3.1. Pathophysiological Mechanisms of CKD-Associated Pruritus

Despite extensive research, the exact pathogenesis of CKD-aP remains elusive and likely multifactorial. No single unified theory fully explains the itch, but contemporary literature highlights several overlapping frameworks from both nephrology and dermatology perspectives. The five most referenced explanatory models are:

#### 3.1.1. Uremic Toxins and Metabolic Factors

Early theories focused on biochemical imbalances such as hyperphosphatemia, hypercalcemia, and elevated parathyroid hormone levels; however, correlations have been inconsistent and correcting these parameters seldom provides sustained itch relief [14]. Increasing attention is directed toward protein-bound, gut-derived uremic toxins (e.g., indoxyl sulfate, p-cresyl sulfate), which accumulate due to inadequate clearance during dialysis and contribute to oxidative stress, inflammation, and neural hyperexcitability [15]. These data indicate that uremic retention plays a contributory but not sufficient role in CKD-aP.

#### 3.1.2. Immune System Dysregulation and Inflammation

A leading contemporary model is that CKD-aP is an inflammatory, immune-mediated pruritus. Studies of skin biopsies and serum in dialysis patients have identified a pro-inflammatory “immunochemical milieu” associated with itch [16]. Notably, patients with CKD-aP show elevated levels of T-helper 1 (Th1) cytokines (interleukin-2, IL-6, interferon-γ, tumor necrosis factor-α) and the itch-specific cytokine IL-31. There is also an increase in dermal mast cells and mast-cell tryptase in itchy patients, suggesting mast cell activation may have a contribution even though histamine levels are not markedly different, indicating a non-histaminergic pathway for itch. The Th1/Th2 balance in skin skews toward Th1 in those with pruritus, reflecting a systemic inflammatory state often present in CKD. This inflammatory hypothesis aligns with clinical findings that “micro-inflammation” correlates with itch severity. It also provides rationale for treatments like thalidomide or corticosteroids, which have shown antipruritic effects by modulating cytokines [17]. Many authors now view CKD-aP as a cytokine-driven chronic itch, where the immune system’s dysregulation in ESRD (possibly due to dialysis membranes, oxidative stress, or comorbid conditions) triggers pruritogenic signaling cascades [18,19]. This model overlaps with the emerging recognition of systemic inflammation as a key player in CKD complications.

Although IL-31 has emerged as a key pruritogenic cytokine in CKD-aP, its precise upstream regulation in uremia remains incompletely defined. Proposed triggers include chronic microinflammation, oxidative stress, and dialysis-related immune activation, yet causal human molecular data are limited. Downstream, IL-31 signaling acts on sensory neurons via IL-31 receptor A and OSMRβ, promoting neuronal sensitization rather than direct histamine release. These pathways are supported primarily by translational and dermatologic models, underscoring the need for human tissue-level validation in CKD populations.

#### 3.1.3. Neuropathic and Neural Mechanisms

Peripheral neuropathy is common in ESRD and affects small unmyelinated C-fibers responsible for itch transmission [20]. Electrophysiologic testing shows decreased neural thresholds and altered conduction in patients with CKD-aP, consistent with heightened peripheral excitability [21]. Chronic stimulation leads to central sensitization, where inhibitory spinal pathways become dysfunctional, contributing to itch persistence independent of peripheral triggers [22]. Improvement following gabapentinoid therapy supports this mechanism [23].

#### 3.1.4. Opioid Receptor Imbalance

Opioid Receptor Imbalance: Another prominent hypothesis is the endogenous opioid dysregulation model of uremic pruritus. In healthy skin, there is a balance between μ-opioid receptors (MOR) and κ-opioid receptors (KOR) that modulates itch and pain: activation of μ-receptors tends to increase itch, whereas activation of κ-receptors relieves itch [24]. In CKD patients, an imbalance in this system has been seen. Skin biopsies from pruritic patients have shown decreased expression of κ-opioid receptors in the epidermis, which could tilt the balance toward a pruritic state. CKD is also associated with elevated endogenous opioids (like β-endorphin) and dysregulation of opioid pathways. This framework gained strong support from clinical trials in recent years, demonstrating that κ-opioid agonists markedly reduce itch in hemodialysis patients [25]. Difelikefalin, a peripherally restricted KOR agonist, was approved in 2021 after Phase III trials showed significant improvement in itch scores and quality of life compared to placebo [26]. Conversely, μ-opioid antagonists (like naltrexone) have also been used off-label with some success in reducing CKD-aP, consistent with this model. The opioid imbalance theory bridges nephrology and dermatology: it involves systemic neurotransmitters and cutaneous nerve receptors. It proposes that excess μ-opioid activity (or deficient κ activity) in CKD patients lowers the itch threshold, and restoring the balance (by blocking μ or stimulating κ) alleviates pruritus. This model does not exclude others—rather, it may work alongside inflammation and neuropathy (for example, inflammation might upregulate MOR expression or downregulate KOR, compounding the itch). The dramatic success of difelikefalin in clinical practice has made the opioid pathway one of the most evidence-backed mechanisms for CKD-aP.

Importantly, opioid receptor imbalance likely does not act in isolation. Experimental data suggest that pro-inflammatory cytokines may modulate MOR and KOR expression on peripheral nerve endings, while chronic inflammation may enhance central neural plasticity, lowering itch thresholds. Thus, opioid dysregulation may represent a downstream amplifier of immune-mediated itch signaling rather than a primary initiating event in all patients.

#### 3.1.5. Xerosis and Skin Barrier Dysfunction

Xerosis is highly prevalent in hemodialysis patients due to reduced sweat and sebaceous gland activity, along with impaired lipid composition of the epidermal barrier [27]. Increased transepidermal water loss and microfissures expose underlying nerve endings to irritants and mechanical triggers [28]. Keratinocyte-mediated inflammatory signaling further links cutaneous and systemic pathways [29]. Xerosis amplifies pruritic perception in patients already sensitized by systemic mechanisms.

Overall, current evidence supports a multifactorial neuro–immuno–cutaneous model of CKD-aP, explaining why many patients require combination therapy to achieve adequate symptom control.

Taken together, available evidence supports a hierarchical neuro–immune–cutaneous model of CKD-aP, in which systemic inflammation and uremic milieu act as upstream drivers, sensitizing peripheral nerves and central itch pathways, while cutaneous barrier dysfunction and opioid imbalance function as modulators of itch intensity. However, mechanistic certainty varies substantially across pathways, with opioid signaling supported by the strongest interventional human data, while cytokine and microbiome-related mechanisms remain largely associative.

### 3.2. Instruments for Assessing Pruritus in CKD Patients

Accurate and standardized assessment of pruritus is essential for evaluating disease burden, monitoring therapeutic response, and enabling correlations between subjective symptoms and underlying molecular or inflammatory mechanisms in CKD-associated pruritus (CKD-aP) [30]. Both unidimensional and multidimensional patient-reported outcome measures (PROMs) have been validated in hemodialysis populations.

Unidimensional intensity scales are widely used due to their simplicity and reproducibility. The Numeric Rating Scale (NRS) and Visual Analog Scale (VAS) quantify itch intensity on numeric or linear scales ranging from “no itch” to “worst imaginable itch” and show strong correlation with each other. Among these, the 11-point NRS—particularly the 24 h Worst Itching Intensity NRS (WI-NRS)—has been extensively validated in CKD-aP and is frequently employed as a primary endpoint in clinical trials, including studies of κ-opioid receptor agonists. A reduction of approximately three points on the NRS is generally considered clinically meaningful [31,32,33]. The Verbal Rating Scale (VRS) provides a categorical alternative (mild, moderate, severe) and is mainly used for rapid clinical screening rather than detailed assessment [34].

While intensity scores capture symptom severity, they do not reflect the multidimensional burden of chronic itch. Therefore, multidimensional instruments are increasingly recommended. The 5-D Itch Scale evaluates itch degree, duration, temporal evolution, functional disability, and body distribution, offering a broader characterization of pruritus and its impact on daily life. It has been validated in hemodialysis patients and is sensitive to treatment-related changes [34].

The Skindex-10 is a CKD-specific quality-of-life instrument focusing on the emotional, symptomatic, and social consequences of pruritus. It has demonstrated excellent internal consistency and strong correlation with itch severity, making it particularly valuable for assessing patient-centered outcomes in both observational studies and interventional trials [35]. Similarly, ItchyQoL provides a more granular evaluation of itch-related symptoms, functioning, and emotional distress, and has been used as a reference instrument in validation studies of newer CKD-specific tools [36].

The Uremic Pruritus in Dialysis Patients (UP-Dial) scale is a recently developed CKD-specific questionnaire that integrates itch intensity, frequency, distribution, and psychosocial impact. It demonstrates high reliability and strong convergent validity with NRS, 5-D Itch, and ItchyQoL, representing a significant advance toward standardized assessment tailored to the CKD population [37].

Single-item measures, such as the pruritus question within the KDQOL-36, remain useful for large epidemiological studies and outcome prediction but lack the depth required for mechanistic or interventional research [38,39].

Overall, unidimensional tools such as NRS are well suited for routine clinical monitoring and trial endpoints, whereas multidimensional instruments (5-D Itch, Skindex-10, UP-Dial) provide a more comprehensive understanding of disease burden and enable meaningful correlations between pruritus severity, quality of life, and molecular or inflammatory biomarkers. Strategic selection of assessment instruments is therefore critical for advancing translational research and mechanism-driven management of CKD-associated pruritus.

### 3.3. Mechanism-Oriented Therapeutic Strategies in CKD-Associated Pruritus

Management of CKD-associated pruritus (CKD-aP) is optimized by aligning therapeutic interventions with dominant pathogenic mechanisms, as multiple pathways may coexist and interact in individual patients [40,41].

Immune- and inflammation-targeted therapies address the upstream inflammatory milieu characteristic of CKD. Narrowband UV-B phototherapy reduces pruritus severity through modulation of cutaneous immune responses, although practical limitations restrict its routine use [41]. Emerging approaches, including IL-31 blockade and JAK–STAT inhibition, aim to disrupt cytokine-driven itch signaling but remain investigational in CKD-aP due to limited human mechanistic data [42].

Neuro-modulatory therapies reflect the contribution of peripheral and central neural sensitization. Gabapentinoids are widely used and demonstrate consistent antipruritic efficacy, supporting a neuropathic component in CKD-aP pathogenesis, though careful dose adjustment is required.

Opioid pathway–targeted therapies currently offer the strongest evidence base. κ-opioid receptor agonists, particularly difelikefalin, selectively attenuate peripheral itch signaling and have shown robust improvements in itch severity and quality of life, leading to regulatory approval for moderate-to-severe CKD-aP. These findings position opioid receptor imbalance as a validated downstream therapeutic target.

Skin barrier–directed interventions, including emollients and urea-based moisturizers, mitigate xerosis-related amplification of itch but are generally insufficient as monotherapy.

Finally, experimental strategies, such as microbiome modulation aimed at reducing gut-derived uremic toxins, represent promising upstream interventions but remain exploratory.

In summary, mechanism-based stratification highlights opioid pathway modulation as the most clinically validated approach, while immune and microbiome-targeted therapies represent evolving avenues with translational potential.

### 3.4. Future Directions

Despite progress, several gaps persist:

- Absence of universally accepted diagnostic and severity criteria,
- Inconsistent implementation of validated PROMs in dialysis settings,
- Insufficient biomarkers for patient stratification,
- Limited diversity and size of study populations.

Advancing these priorities will support early identification and personalized treatment of CKD-aP, reducing long-term symptom burden and improving patient outcomes.

## 4. Conclusions

Chronic kidney disease-associated pruritus is increasingly recognized as a systemic, mechanism-driven condition rather than a secondary dermatologic symptom. Current evidence supports a neuro–immune–cutaneous model in which systemic inflammation and uremic milieu initiate pruritogenic signaling, neural sensitization and opioid receptor imbalance amplify itch perception, and skin barrier dysfunction modulates symptom severity.

Among proposed pathways, opioid receptor dysregulation is supported by the strongest translational and interventional human data, exemplified by the clinical efficacy of κ-opioid receptor agonists. In contrast, cytokine-mediated and microbiome-related mechanisms, while biologically plausible, remain incompletely defined and warrant further molecular validation in CKD-specific settings.

Integrating mechanistic insights with validated patient-reported outcome measures may enable improved patient stratification and facilitate the development of targeted, mechanism-based therapies. Continued interdisciplinary research is essential to translate emerging molecular concepts into effective, personalized management strategies for patients undergoing hemodialysis.

## Data Availability

No new data were created or analyzed in this study. Data sharing is not applicable to this article.

## Supplementary Materials

The following supporting information can be downloaded at: https://www.mdpi.com/article/10.3390/ijms27020851/s1. Reference [43] is cited in the supplementary materials.
